# Human lung development: recent progress and new challenges

**DOI:** 10.1242/dev.163485

**Published:** 2018-08-15

**Authors:** Marko Z. Nikolić, Dawei Sun, Emma L. Rawlins

**Affiliations:** 1 Wellcome Trust/CRUK Gurdon Institute, Wellcome Trust/MRC Stem Cell Institute, Department of Pathology, University of Cambridge, Cambridge CB2 1QN, UK; 2 University of Cambridge School of Clinical Medicine, Department of Medicine, Cambridge CB2 0QQ, UK

**Keywords:** Bronchi, Alveolar, ESC, iPSC, Progenitor, Stem cell, Lung disease

## Abstract

Recent studies have revealed biologically significant differences between human and mouse lung development, and have reported new *in vitro* systems that allow experimental manipulation of human lung models. At the same time, emerging clinical data suggest that the origins of some adult lung diseases are found in embryonic development and childhood. The convergence of these research themes has fuelled a resurgence of interest in human lung developmental biology. In this Review, we discuss our current understanding of human lung development, which has been profoundly influenced by studies in mice and, more recently, by experiments using *in vitro* human lung developmental models and RNA sequencing of human foetal lung tissue. Together, these approaches are helping to shed light on the mechanisms underlying human lung development and disease, and may help pave the way for new therapies.

## Abstract (precis)

**Summary:** This Review describes how recent technological advances have shed light on the mechanisms underlying human lung development and disease, and outlines the future challenges in this field.

## Introduction

Lung cancer is the most common cancer worldwide, and end-stage respiratory failure accounts for the third highest cause of mortality due to non-infectious disease (Global status report on noncommunicable diseases 2010, World Health Organization; http://www.who.int/nmh/publications/ncd_report2010/en/). The mortality is partly due to irreversible destruction of lung tissue and the inability to meet the demands for transplantation. Lung transplantation itself is a high-risk procedure that has a 5-year survival rate of only 54%, partly due to immune rejection (Thabut and Mal, 2017). Characterising the different progenitor populations in the developing human lung is thus an essential part of regenerative medicine, particularly as their therapeutic potential may differ from that of adult stem cells. If in the future we are to help patients to regenerate lung tissue, then we need to understand in detail how the human lung develops and, in particular, how the various developing cell populations contribute at a molecular and cellular level to the creation of such a complex organ. This will also aid our understanding of how other endodermal organs develop, particularly those that share comparable developmental mechanisms.

Beyond regenerative medicine, the study of human lung development is important for understanding disease mechanisms. It is immediately obvious that human developmental biology will provide insight into lung conditions experienced by premature neonates whose lungs are still going through embryonic developmental stages at the time of birth (Surate Solaligue et al., 2017). However, a recent study revealed the surprising finding that some of the most tightly linked genetic variants that predispose a person to chronic adult lung disease were predicted to function in lung development (Hobbs et al., 2017). This strongly suggests that developmental events have life-long consequences for respiratory health. Recently, a large study that examined lung structure in >3000 individuals reported that developmental variation in human lung branching, specifically a central airway branch variation, is associated with chronic obstructive pulmonary disease (COPD) (Smith et al., 2018). Moreover, one of these airway branch variations is associated with genetic polymorphisms within the *FGF10* gene, which is known to be crucial for branching morphogenesis in the developing lung (Bellusci et al., 1997; Danopoulos et al., 2018; Park et al., 1998; Peters et al., 1994).

Advances in human developmental biology may also be directly applied to treat disease. The discovery of induced pluripotent stem cells (iPSCs) derived from human fibroblasts (Takahashi and Yamanaka, 2006) opened the door to patient-specific disease modelling. iPSCs can be derived from any somatic cell – typically skin or blood – and differentiated into any cell type of interest for disease modelling and drug screening. This technology also brings us a step closer to personalised cell-based therapies. Research on murine lung development has been crucial in providing a developmental roadmap to direct the stepwise differentiation of iPSCs into lung epithelial cells (Swarr and Morrisey, 2015). However, only recently have equivalent studies been performed using human embryonic lung tissue to allow iPSC differentiation attempts to be further improved and adequately validated (Miller et al., 2017; Nikolić et al., 2017).

In this Review, we summarise our current knowledge of human lung development, highlighting areas of similarity to and divergence from mouse biology. We also discuss recent advances in the available human *in vitro* model systems and how these are already providing insights into developmental mechanisms. Finally, we explore future challenges and important out-standing questions for the field, with a focus on the technological hurdles, such as validation of experimental systems and scale-up of cell production, that must be overcome in order to move towards the clinic.

## An introduction to human lung development

### The human adult lung

**Fig. 1.**
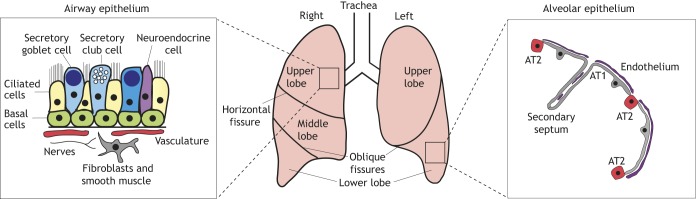
**Human adult lung structure and cell types.** Lobular structure of the human adult lung. Insets depict the cell types found within the airway epithelium (left) and the alveolar epithelium (right).

The lungs are a complex structure of branched airways and blood vessels that unite at the most distal part, the alveoli, for gas exchange. They are found on either side of the heart and in humans have three right and two left lobes (Fig. 1), with the bottom of the lungs resting over the concave-shaped diaphragm (Drake et al., 2014). Both lungs are surrounded by a membrane known as the pleura, which is referred to as the mesothelium in mouse (Hogan et al., 2014; Morrisey and Hogan, 2010). The most proximal airway, the trachea, divides at the carina forming the left and right main stem bronchi. Each main bronchus divides further into secondary, or lobar, bronchi and subsequently into progressively narrower airways until the smallest bronchioles connect to the alveoli. Bronchi are reinforced with hyaline cartilage in order to maintain airway patency, whereas bronchioles are surrounded by smooth muscle. Air is transported through the airways all the way to the alveoli, where gas exchange takes place between the thin alveolar epithelial cells and the fine capillary network that covers them (Weibel, 1963).

### Human adult lung cell types

The various cell types found in human lungs can be categorised into epithelium, endothelium (vasculature and lymphatics), pleura/mesothelium, airway and vascular smooth muscle, pericytes, fibroblasts, neurons and immune cells such as alveolar macrophages. Many of these cell types can be further classified based on their position along the epithelial branching tree. Generally accepted lung cell type markers are listed in Table 1, although many of these are not absolutely specific for a single lung cell type.

**Table 1.**
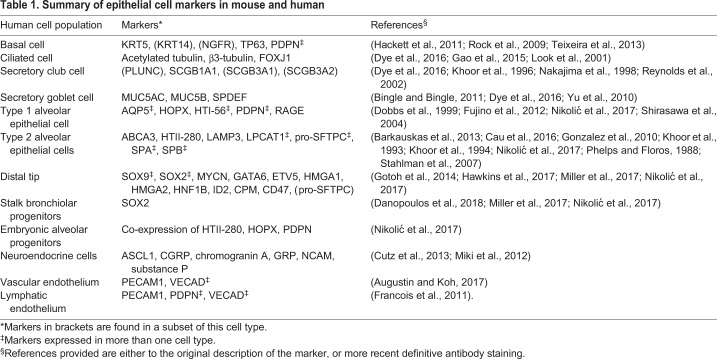
**Summary of epithelial cell markers in mouse and human**

#### Airway cell types

Lung epithelial cells are broadly subdivided into airway (tracheal/bronchiolar) and alveolar types. The human tracheobronchial airways are lined by pseudostratified epithelium in which each cell makes contact with the basement membrane. Below the basement membrane are blood and lymphatic vessels, smooth muscle, cartilage, fibroblasts and nerves (Hogan et al., 2014). The height of the airway lining and the proportion and density of the different cell types vary along the proximal-distal axis of the airways (Mercer et al., 1994). In the mouse trachea, there is a similar basic organisation of pseudostratified mucociliary epithelium and underlying mesenchyme, whereas lower mouse airways have a simple columnar epithelium (Hogan et al., 2014).

The conducting airway epithelia consist mostly of basal, secretory (club, mucous and serous subtypes) and ciliated cells (Fig. 1). Together, these cells comprise the mucociliary escalator, so called because it transports inhaled particles trapped in the mucus up and out of the airways. In both human and mouse adult airways, basal cells are stem cells that self-renew and differentiate into secretory and ciliated cells during homeostasis and repair (Evans et al., 2001; Hong et al., 2004; Rock et al., 2009; Teixeira et al., 2013; Watson et al., 2015). Basal cells are present throughout human conducting airways, but are confined to the trachea and primary bronchi of mice (Boers et al., 1998; Nakajima et al., 1998). In mice, it is clear that the dome-shaped club cells in the bronchioles act as stem cells for day-to-day airway epithelial maintenance and repair (Giangreco et al., 2009, 2002; Hong et al., 2001; Rawlins et al., 2009b) and can even contribute to the alveolar epithelium following injury (Guha et al., 2017). In contrast, club cells within the pseudostratified mouse trachea are progenitors that do not exhibit long-term self-renewal, but do divide for a limited time and can produce new ciliated cells (Rawlins et al., 2009b; Watson et al., 2015). Mouse tracheal club cells can de-differentiate to a basal stem cell phenotype if the endogenous basal cells are killed experimentally (Tata et al., 2013). In human lungs, the secretory cells are predominantly of the mucous subtype (Mercer et al., 1994). It is not clear whether mucous-secreting cells retain the ability to proliferate and function as stem/progenitor cells, or indeed if human club cells can do so (Teixeira et al., 2013). In mice, ciliated cells are terminally differentiated and do not proliferate even after injury (Pardo-Saganta et al., 2013; Rawlins and Hogan, 2008; Rawlins et al., 2007); it is likely that they behave similarly in humans.

#### Minor lung epithelial cell types

Rare cell types found in the airways include brush cells and pulmonary neuroendocrine cells (PNECs). Brush cells make up less than 1% of the airway epithelium and have recently been shown to have a chemosensory role that may allow them to detect bacterial infections (Krasteva et al., 2011, 2012; Tizzano et al., 2011). PNECs are hypoxia-sensitive cells (Cutz et al., 2013). In the mouse trachea, PNECs are rare, solitary cells that are derived from basal cells (Watson et al., 2015). Lower down the mouse airways, PNECs are clustered into groups of up to 30 cells, known as neuroendocrine bodies (NEBs), which are located preferentially at airway branch points and have been characterised as a putative stem cell niche (Guha et al., 2012; Reynolds et al., 2000). Careful morphometric studies in humans have shown that PNECs make up less than 1% of airway epithelial cells and are mostly solitary, but can be observed in clusters of six to eight cells (Gosney, 1993). Interestingly, although the percentage of PNECs in the airways does not change with age in humans, the number of NEB clusters is greatest during the foetal stage and in young adults (Cutz et al., 1985; Gosney, 1993). Additional rare airway stem cell types that can be activated following severe injury have been identified in mice, but not yet in humans. These include the putative bronchoalveolar stem cells (Kim et al., 2005; Lee et al., 2014) and rare basal-like cells of the distal airways (Kumar et al., 2011; Vaughan et al., 2015; Xi et al., 2017; Yang et al., 2018; Zuo et al., 2014). Increasing numbers of studies in mice are now showing that communication between the airway epithelium and underlying mesenchymal cells is required for normal homeostatic maintenance and a proliferative response to injury (Lee et al., 2017; Volckaert et al., 2011; Volckaert et al., 2017).

#### Submucosal gland cell types

Submucosal glands are continuous with the airway epithelium and are located below the luminal surface. They secrete mucous and other substances that help protect the lungs from particles and infectious agents (Liu et al., 2004). In humans, and other large mammals, submucosal glands are found in all of the cartilaginous airways, whereas in mice they are restricted to the first few cartilage rings of the proximal trachea (Borthwick et al., 1999; Innes and Dorin, 2001; Meyrick et al., 1969). The submucosal gland epithelium contains mucous- and serous-secreting cells, as well as myoepithelial basal cells. In mice, the submucosal myoepithelial basal cells function as stem cells for the submucosal gland itself and also as reserve stem cells that contribute to regeneration of the surface epithelium of the airways after severe injury (Lynch et al., 2018; Tata et al., 2018a). Interestingly, pig submucosal glands also contribute to the airway surface epithelium following injury, strongly suggesting that the situation in the human lung will be similar (Tata et al., 2018a).

#### Alveolar cell types

The alveolar epithelium (Fig. 1) consists of type I and type II alveolar cells (AT1 and AT2 cells) that are surrounded by capillaries and fibroblasts (Herzog et al., 2008; Weibel, 2015; Williams, 2003). AT1 cells are flat, highly extended and specialised for gas exchange as they cover more than 95% of the gas exchange surface area. AT2 cells are cuboidal, more common, and are specialised for the production of surfactant – a complex mixture of proteins and phosopholipids that decreases surface tension in the alveolar region (Crapo et al., 1982; Hogan et al., 2014; Weibel, 2015; Williams, 2003). AT2 cells are the major alveolar epithelial stem cell as they can both self-renew and differentiate into AT1 cells (Barkauskas et al., 2013; Desai et al., 2014; Rock et al., 2011a). Recent reports have characterised a subpopulation of mouse AT2 cells that preferentially act as the alveolar stem cell during steady-state turnover (Nabhan et al., 2018; Zacharias et al., 2018) and also suggest that a similar subpopulation can be identified in adult human alveoli. Adult rodent and human AT1 cells are characterised by a limited proliferative capacity following injury *in vivo* and can de-differentiate to AT2-like cells when cultured *in vitro* (Danto et al., 2012; Evans et al., 1973; Hogan et al., 2014). There are multiple populations of mouse alveolar fibroblasts, some of which interact with the epithelium to maintain normal homeostasis (Lee et al., 2017; Zepp et al., 2017). Traditionally, alveolar fibroblasts have mostly been characterised as myofibroblasts and lipofibroblasts but their exact roles are not yet defined and there is even controversy about the existence of lipofibroblasts in human lungs (El Agha et al., 2017; McCulley et al., 2015; Rehan et al., 2006). The lung also contains a resident population of immune cells, alveolar macrophages, that have important functions in surfactant homeostasis and innate immunity (Bhattacharya and Westphalen, 2016).

#### Vascular cells

Blood vessels are an integral part of the lung both in the airway and, especially, in the alveolar gas exchange region. The cellular composition of the vasculature in terms of associated smooth muscle, pericytes and other fibroblasts depends on the exact location within the lung (Kool et al., 2014). Rather than just delivering oxygen and nutrients, endothelial cells lining the vessels also modulate blood coagulation, transport of inflammatory cells and epithelial homeostasis and repair (Rafii et al., 2016).

### Stages of human lung development

**Fig. 2.**
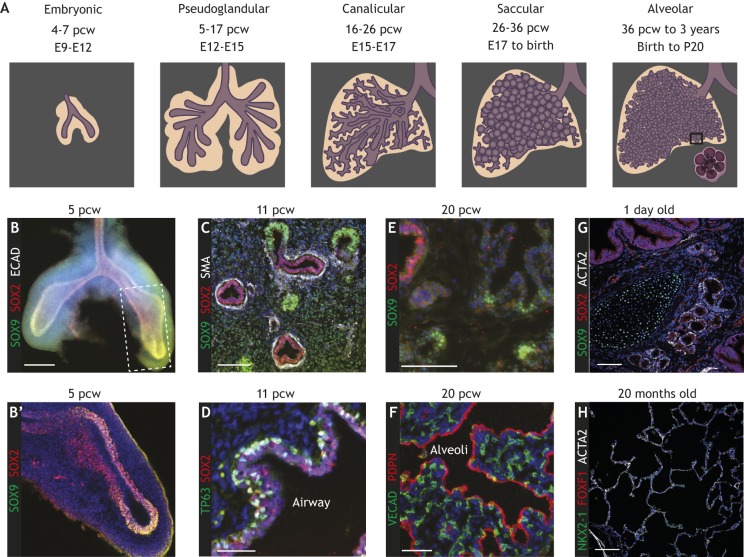
**Stages of human lung development.** (A) Schematics depicting general lung morphology across the five different stages of human lung development: embryonic, pseudoglandular, canalicular, saccular and alveolar. For each stage, the developmental period is indicated, for human in post-conception weeks (pcw) and for mouse in embryonic days (E) and postnatal days (P). Boxed area is enlarged to show the gas exchange occurring across the alveolar epithelium. (B,B′) Cryosection of an embryonic stage lung showing the primary branches and SOX2/SOX9 co-expression in the tips; the boxed area is magnified in B′. (C,D) Cryosections of pseudoglandular stage lungs showing ongoing tip SOX2/SOX9 co-expression and airway differentiation as indicated by the expression of smooth muscle actin (SMA) (C, white), which marks smooth muscle cells, and TP63 (D, green), which marks differentiating basal cells. (E,F) Cryosections of canalicular stage lungs showing SOX9^+^/SOX2^−^ distal tips (in E). Alveolar differentiation is initiated at this stage, as indicated by the widening alveolar spaces. Proximity to developing vasculature, as marked by VE-cadherin (VECAD; CDH5) (green) and podoplanin (PDPN, red) is illustrated in F. Note that there is a gap in the available images at the saccular stage where distal tips are presumed to disappear and alveolar differentiation progresses. (G,H) Cryosections of alveolar stage postnatal lungs, showing the expression of SOX9 (cartilage, green), SOX2 (airway cells, red) and ACTA2 (smooth muscle, white) in G, and NKX2-1 (lung epithelium, green), FOXF1 (mesenchyme) and ACTA2 (smooth muscle, white) in H. At this stage, SOX9^+^ distal tips are no longer seen (G), but there has been continued growth and septal formation to make alveoli (H). Images in A-F are reproduced from Nikolić et al., 2017. Images in G and F were kindly provided by Jeff Whitsett, University of Cincinnati College of Medicine (https://research.cchmc.org/lungimage/). Scale bars: 200 μm (B); 50 μm (C,D,F); 100 μm (E,G,H).

Human lung development is divided into different morphological stages (Fig. 2A) that correspond to key developmental transitions: (1) embryonic, (2) pseudoglandular, (3) canalicular, (4) saccular and (5) alveolar (Burri, 1984; Rackley and Stripp, 2012). The timing of the stages is anecdotally said to be overlapping due to non-synchronous development of the lung, although there is likely to be more inherent variation (either environmental or genetic) in human development compared with laboratory animals. Standard human embryo staging systems are typically used (Hern, 1984; O'Rahilly and Muller, 1987), but there may also be some technical variation. As is the case in mice, the human lung epithelium is derived from the endoderm whereas the surrounding mesenchyme derives from the mesodermal germ layer (Burri, 1984).

The embryonic phase of human lung development (Fig. 2B,B′) spans approximately 4-7 post-conception weeks (pcw). During this time, the primary left and right lung buds appear from the foregut endoderm towards the end of week 4 and rapidly undergo branching to set up the overall lobular structure of the lung by the end of week 5.

The pseudoglandular phase occurs from approximately 5 to 17 pcw (Fig. 2C,D). During this period, the lung continues to grow by branching morphogenesis, the airway tree is laid down and begins to differentiate with cartilage, and the smooth muscle and mucous glands are already visible. It is likely that human airway branching occurs mostly dichotomously (Isaacson et al., 2017). Studies in mice have suggested that the epithelial branching pattern is relatively stereotypic at this stage, although work in humans shows that there is comparably more variation (Metzger et al., 2008; Smith et al., 2018). Foetal breathing movements begin by 10-11 pcw and are hypothesised to play a role in lung growth. Blood vessel development occurs concurrently with epithelial branching, and vessels run alongside the airways but branch more slowly (deMello and Reid, 2000). At the end of the pseudoglandular stage, the complete structure of the human airway tree has been laid down (Kitaoka et al., 1996) and airway epithelial differentiation is progressing (Khoor et al., 1996).

The canalicular phase spans 16-26 pcw (Fig. 2E,F). It is estimated that three further rounds of epithelial branching occur during this stage in order to produce the future alveolar regions. Moreover, existing airways continue to increase in size and the most distal epithelial tubes – the future alveoli – widen into the airspaces and their surrounding mesenchyme thins. The capillary networks come into close proximity to the distal epithelial airspaces (Fig. 2F) (deMello and Reid, 2000) and the first morphological signs of alveolar epithelial cell differentiation (a decrease in columnar height) occur.

The saccular stage ranges from about 24 to 38 pcw and coincides with the end of branching morphogenesis. The distal airspaces now appear as thin-walled terminal saccules that cluster at the ends of the airways. These further increase in size and become completely wrapped in a capillary bilayer, which appears to be formed as the capillaries that surround each saccule are pushed together as they expand (Burri, 1984). Alveolar epithelial differentiation continues and, in particular, the surfactant system of the AT2 cells matures and lamellar bodies – specialised organelles for the production and recycling of surfactant – and surfactant secretion can be detected (Khoor et al., 1994; Oulton et al., 1980; Stahlman et al., 2007).

The alveolar stage refers to the process of alveolar formation during which septae grow from the saccular walls to subdivide the distal saccules into alveoli, thus increasing the surface area for gas exchange (Fig. 2G,H). In parallel, microvascular maturation occurs and the double capillary network observed at the saccular stage fuses into a single capillary system, meaning that each capillary is completely surrounded by gas exchange surfaces (Schittny, 2017). Alveolar formation has typically been considered to occur from 36 weeks up to 3 years after birth. However, the use of new imaging technologies and detailed stereology suggest that alveolar formation continues into young adulthood (∼21 years) (Herring et al., 2014; Narayanan et al., 2012). Prior to birth, the lung epithelium expresses high levels of chloride channels and is a net secretor of fluid into the amniotic cavity. At the time of birth, however, there is a rapid switch to sodium, and hence water, absorption triggered by β-adrenergic signalling.

### The molecular regulation of human lung development

The molecular regulation of mouse lung development has been extensively studied (Cardoso and Lu, 2006; Swarr and Morrisey, 2015). By contrast, very little information on the molecular regulation of human lung development is currently available. A key line of investigation has been the cloning of mutant genes from human patients with congenital lung disease and mechanistic investigation of gene function using genetically altered mice. For example, the transcription factor Nkx2-1 is the first factor to be expressed in the region of the embryonic foregut endoderm that will bud into the lung (Ikeda et al., 1995; Lazzaro et al., 1991). Mouse *Nkx2-1* mutants fail to make lungs and Nkx2-1 has been shown to be required throughout mouse lung development and in the adult (Minoo et al., 1999; Tata et al., 2018b). Mutations in *NKX2-1* are associated with congenital lung disease, leading to the hypothesis that NKX2-1 plays very similar mechanistic roles in mouse and human lung development (Devriendt et al., 1998). Similarly, genes that are mutated in human neonatal respiratory distress syndrome, such as those encoding surfactant proteins B and C, ABCA3 and the transcription factor FOXM1, have been shown to play key roles in alveolar development in mice (Whitsett, 2014). The genetic manipulation of human adult airway epithelial cell cultures has also been used to identify transcription factors that are required for ciliated cell differentiation, and these are largely assumed to also function in the development of human embryonic airway ciliated cells (Boon et al., 2014; Wang et al., 2018). However, until recently, experimental systems that allow a mechanistic investigation of the molecular regulation of human embryonic lung development have been available to only very few laboratories and progress has consequently been limited.

## Studies of human lung development using human lung tissue

Studying human lung development, especially late-stage development, is hindered by limited access to human foetal lungs. Specifically, the lower limit of neonatal viability is approximately 23-24 pcw and in most countries human foetuses beyond ∼20 pcw are not available for research. Although morphological analyses of human embryonic and foetal lungs have been extensive, modern molecular techniques have not been widely applied and the embryonic mouse lung has thus been used as a substitute for studying human lung development. However, recent advances in RNA sequencing (RNA-seq) and single cell-based approaches have allowed more detailed characterisations of human lung tissue to be performed.

**Fig. 3.**
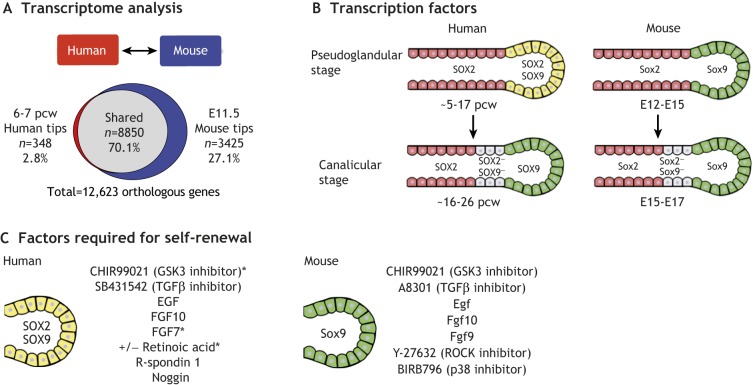
**Selected differences between mouse and human lung development.** (A) Comparison of human and mouse distal epithelial tip transcriptomes reveals shared and unique transcripts (Nikolić et al., 2017). (B) One specific example of a molecular difference between mouse and human lungs is that SOX2 expression extends to the distal epithelial tip in pseudoglandular stage human lungs, but not in mouse. However, by the canalicular stage, human and mouse lungs have similar SOX2 expression in the differentiating airway only. (C) Mouse (Nichane et al., 2017) and human (Miller et al., 2017; Nikolić et al., 2017) distal epithelial tip progenitors are maintained in culture via the activation, or inhibition, of different signalling pathways. Asterisks indicates factors reported by Miller et al., 2017 that were needed in addition to those reported by Nikolić et al., 2017 for the self-renewal of human epithelial distal tip progenitors.

RNA-seq, for instance, has been used to characterise global gene expression changes throughout whole human lungs in a developmental time course (Bernstein et al., 2010; Feng et al., 2014; Kho et al., 2016). More recently, comparative RNA-seq between human foetal organs at ∼6-8 pcw was used to generate organ-specific, including lung-specific, transcriptional signatures (Gerrard et al., 2016). Cell type-specific transcriptome analysis has also been performed on microdissected branching epithelial tip cells from human pseudoglandular stage lungs by RNA-seq (Miller et al., 2017; Nikolić et al., 2017). A genome-wide comparison of the transcriptome of human epithelial tip cells with previously published mouse tip microarray data (Laresgoiti et al., 2016) has shown that 96% of orthologous genes expressed in human tips are also present in mouse (Nikolić et al., 2017) (Fig. 3A). However, multiple subtle differences between mouse and human were found. For example, *BMP2* and *BMP7* are highly expressed in human tips compared with *Bmp4* in the mouse (Bellusci et al., 1996). These data suggest that the human tip epithelium is analogous to the mouse population with a highly conserved transcriptome, but that the differences are likely to be functionally significant.

During mouse lung development, distal tip epithelial cells are Sox9^+^/Id2^+^ and act as multipotent progenitors by generating first bronchiolar, and then alveolar, descendants (Alanis et al., 2014; Rawlins et al., 2009a). During the mouse pseudoglandular stage, cells that exit the distal tip turn off Sox9, upregulate Sox2 and differentiate along bronchiolar lineages. Hence, there is always a clear demarcation between Sox9^+^ tip cells and Sox2^+^ stalk bronchiolar progenitors, and this has been used routinely for assessing mouse mutant phenotypes and for validation of human pluripotent stem cell differentiation (Fig. 3B). By contrast, in human lungs SOX2 is consistently co-expressed with SOX9 in tip cells throughout the pseudoglandular stage (Danopoulos et al., 2018; Miller et al., 2017; Nikolić et al., 2017). As human cells exit the tip progenitor domain and start to differentiate along airway lineages, they turn off SOX9, but retain SOX2 (Fig. 3B). The function of SOX2 in the human epithelial tip progenitors is currently unknown, but this result illustrates one of the molecular differences between human and mouse lungs and also shows that SOX2 expression alone cannot be used as a marker for human bronchiolar progenitors.

The morphological and molecular differences between human and mouse lungs strongly suggest that certain aspects of human lung development can only be studied using human cells. One experimental approach has been to grow late-stage (∼20 pcw) distal human lung epithelium in 2D as a model for AT2 and AT1 cell differentiation. Such studies have led to important molecular insights, for example elucidating the roles of glucocorticoid signalling (Mendelson et al., 1997; Mishra et al., 2018). The culture of human foetal lung explants has also allowed the effects of specific signalling activators and inhibitors on cell fate, proliferation and fluid secretion to be studied (Brennan et al., 2016; Haitchi et al., 2009; Han et al., 2003; Rajatapiti et al., 2010). However, the major limitations of these organ cultures are their inaccessibility to modern genetic techniques and the need for a continuous supply of fresh tissue. Organoid cultures have recently been grown from human distal epithelial tips by initially expanding self-renewing tip cells in Matrigel and subsequently differentiating them into an alveolar or bronchiolar fate (Fig. 3C) (Miller et al., 2017; Nikolić et al., 2017). The self-renewing phase of these cultures can be maintained long-term (without introducing karyotypical abnormalities), freeze-thawed and should be amenable to genetic manipulation (Broutier et al., 2016). A corresponding study using the equivalent population of mouse epithelial tip cells also showed successful tip self-renewal and subsequent production of airway and alveolar cell types, although a different combination of growth factors and inhibitors was reported (Fig. 3C) (Nichane et al., 2017). These differing media requirements for growth illustrate that the molecular differences observed between human and mouse embryonic lungs are indeed functionally relevant. One limitation of the human lung embryonic organoid cultures so far is that they have not yet been demonstrated to produce mature, differentiated cell lineages, although this problem is likely to be solved in the future.

**Fig. 4.**
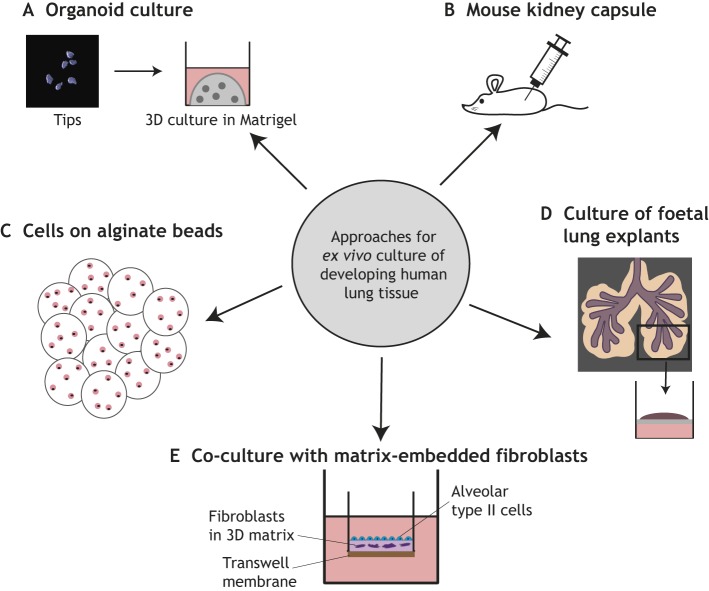
**Methods for *ex vivo* culture of developing human lung tissue.** (A) Culture set-up for distal tip organoids. Whole distal epithelial tips are placed within Matrigel and growth medium is added on top. (B-D) Other available culture systems for human developing lungs and cells include immune-compromised mouse kidney capsule grafting (B), seeding cells on alginate beads in bioreactors (C), culture of lung explants floating at the surface (or submerged within) growth medium (D) and co-culture with matrix-embedded fibroblasts (E).

The saccular/early alveolar period of human lung development after 20 pcw is still largely unexplored, mainly owing to the lack of tissue for analysis. Modern neonatal intensive care strategies can allow premature neonates to survive from ∼23 pcw, although these children often suffer from long-term respiratory conditions as a result of lung immaturity at birth and the effects of the therapies that kept them alive. Therefore, another approach for studying human foetal lung development has been to mature tissue, or cells, from <20 pcw lungs to a developmental stage at which the lung pathologies affecting premature neonates can be modelled (Fig. 4). For example, human foetal mesenchymal 3D organoid cultures based on alginate beads have been used to study cell-cell interactions and the effects of changes in oxygen levels for the purposes of modelling idiopathic pulmonary fibrosis (IPF) and bronchopulmonary dysplasia (BPD) (Sucre et al., 2016; Wilkinson et al., 2017). However, late foetal epithelial and vascular development is still very under-characterised. A recent development has been to maintain human foetal AT2 cells for up to 7 days *in vitro* by using organotypic co-cultures with matrix-embedded fibroblasts (Sucre et al., 2018b). Another approach has been to transfer human foetal lung pieces from ∼11-15 pcw into the kidney capsule of immune-compromised mice for further maturation. This kidney capsule technique has allowed the maturation of tissue samples to a much greater degree than can be obtained by *in vitro* organ culture (De Paepe et al., 2012; Maidji et al., 2012; Pavlovic et al., 2008). For example, some alveolar morphogenesis has been achieved, which could allow the cellular mechanisms of microvascular development to be investigated (De Paepe et al., 2012). Kidney capsule-grafted lung fragments have also been used as a model of congenital human cytomegalovirus infection (Maidji et al., 2012).

Lung development continues after birth as the lung grows; new alveoli are formed and the microvasculature is remodelled (Schittny, 2017). Studies using postnatal developing lungs are, again, restricted by lack of material. Efforts have been made to examine lung morphology, molecular and global transcriptional changes in the lungs of infants who have died with lung pathologies, compared with non-lung pathologies (Bhatt et al., 2001; Bhattacharya et al., 2012; Sucre et al., 2018a). Such work is now bringing insight into the molecular mechanisms of both postnatal lung development and lung pathologies. For example, it has long been established that *Pdgfa^−/−^* mouse lungs have a failure of alveolar septation and low levels of elastin caused by the loss of elastin-producing myofibroblasts (Boström et al., 1996). Recently, it was shown that there is reduced PDGFRα expression in human lung fibroblasts from ventilated pre-term infants and that attenuated PDGF signalling independently contributes to defective septation in a mouse model of BPD (Oak et al., 2017; Popova et al., 2014). The challenges of studying the molecular and cellular mechanisms of postnatal alveologenesis prompted the formation of the collaborative LungMAP consortium (Ardini-Poleske et al., 2017). Their efforts include detailed molecular and structural analyses of human lung development from 23 pcw to ∼10 years, and human postnatal imaging, transcriptomics and proteomics data are gradually being released via their website (www.lungmap.net). This will likely lead to new mechanistic insights into this under-explored developmental phase.

## Studies of human lung development using pluripotent stem cells

Pluripotent stem cells (PSCs) can be derived from the inner cell mass of the early embryo (in the case of embryonic stem cells, ESCs), or can be reprogrammed from fully differentiated cells (in the case of iPSCs). They retain the potential to differentiate into every cell type of the body. Using human PSC differentiation to mimic human lung development has provided a powerful tool for understanding human organogenesis, and human (h)PSC-derived lung cells are already being used for disease modelling and drug screening (see Box 1). Moreover, iPSCs provide a gene-correctable cell source that may be useful for autologous transplantation, minimising the need for immunosuppression, although this remains a controversial hypothesis (Liu et al., 2017).

Box 1. The use of hPSCs for pulmonary disease modelling When fully optimised, PSC-derived models of pulmonary disease are likely to be very useful for modelling the progression of genetic diseases for which only end-stage samples are normally available. They could also provide large numbers of disease-phenotype, and control, lung cells for therapeutic discovery and toxicology testing. Proof-of-principle pulmonary disease modelling has already been performed using hPSC-derived lung cells. For example, iPSC-derived airway epithelial organoids have been produced from cystic fibrosis patients and shown to mimic known *in vitro* disease phenotypes (Dekkers et al., 2013; McCauley et al., 2017). Similarly, iPSC-derived alveolospheres from children with surfactant protein B mutations develop the observed *in vivo* surfactant processing phenotypes (Jacob et al., 2017). iPSC-derived alveolospheres have also been shown to recapitulate the *in vivo* phenotype of swollen lamellar bodies when treated with the drugs amiodarone or GNE7915, suggesting that such cells could be highly useful for toxicology testing (Yamamoto et al., 2017). Finally, it may be possible to model more complex conditions using hPSC differentiation protocols that result in lung organoids containing both epithelial and mesenchymal cell types. Such organoids have been successfully infected with respiratory syncytial virus and recapitulate the epithelial shedding observed in the early stages of the disease, although the lack of immune cells may be a limitation here (Chen et al., 2017). Moreover, a genetic form of pulmonary fibrosis was partially recapitulated in hPSC-derived lung organoids and the data obtained suggested that epithelial damage was a key driver of the disease (Chen et al., 2017).

### A developmental roadmap for lung differentiation

**Fig. 5.**
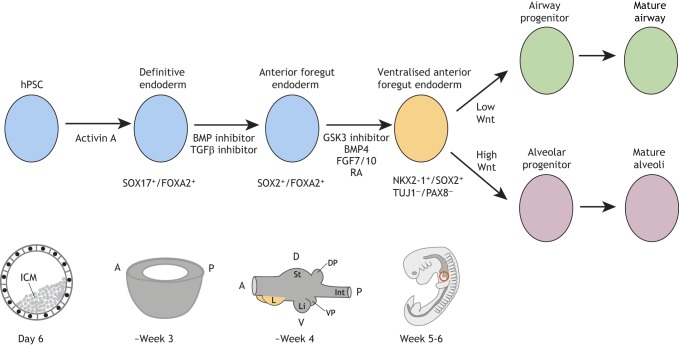
**Studying human lung development using hPSCs.** The usual strategy for differentiating human pluripotent stem cells (hPSCs) to lung cells relies on maturing the cells through sequential progenitor stages that correspond, as closely as possible, to normal embryonic development. Typically, hPSCs, which are equivalent to the cells found in the inner cell mass of the blastocyst (ICM, day 6 post-conception), are differentiated to SOX17^+^/FOXA2^+^ definitive endoderm (∼3 pcw, equivalent to E7.5 in mouse). These cells are then matured to SOX2^+^/FOXA2^+^ anterior foregut endoderm (∼4 pcw, equivalent to E8.5-E9.5 in mouse) from which the lungs bud. Further differentiation proceeds via a NKX2-1^+^/SOX2^+^ ventralised anterior foregut endoderm that corresponds to the NKX2-1^+^ lung foregut progenitor domain characterised in mice (5-6 pcw). NKX2-1 is known to be also expressed in the developing brain and thyroid, hence TUJ1^−^/PAX8^−^ marker selection is also used to define NKX2-1^+^ lung progenitor cells *in vitro*. Current hPSC stepwise differentiation protocols sort a pure population of NKX2-1^+^/SOX2^+^/TUJ1^−^/PAX8^−^ lung progenitor cells, which are subsequently differentiated towards airway or alveolar fate by modulation of Wnt signalling. Key signalling pathways are depicted with a more detailed summary in Table S1. A, anterior; D, dorsal; DP, dorsal pancreas; Int, intestine; L, lung; Li, liver; P, posterior; RA, retinoic acid; St, stomach; V, ventral; VP, ventral pancreas.

Stepwise differentiation strategies that mimic the *in vivo* signalling pathways driving human lung development have been developed and adopted for the *in vitro* differentiation of hPSCs. Such protocols have so far been almost exclusively based on mouse development due to the lack of available human data. hPSCs are matured through various developmental stages, including definitive endoderm, anterior foregut endoderm and ventralised anterior foregut endoderm (Fig. 5). This results in a mixed population of ventral anterior foregut endoderm-like cells, including lung lineage cells that are positive for *NKX2-1* and *SOX2*, but negative for *TUJ1* (*TUBB3*; neuronal lineage) and *PAX8* (thyroid lineage).

Anterior foregut endoderm progenitors were first efficiently generated from definitive endoderm by systematically screening for conditions that activate *SOX2* (a marker of the foregut) and repress *CDX2* (a marker of the hindgut). These progenitors could be further induced to express surfactant protein C (SFTPC), a distal alveolar progenitor marker (Green et al., 2011). Subsequently, NKX2-1^+^ lung progenitors were generated using similar stepwise induction methods and then differentiated towards the major proximal lung cell fates (Mou et al., 2012; Wong et al., 2012). These studies pioneered *in vitro* pulmonary lineage specification. However, we note that, in general, these methods generate a very low percentage of NKX2-1^+^ lung progenitors and very few specific bronchiolar, or alveolar, lineage cells (see Table S1). Moreover, there is extensive variability between hPSC cell lines in terms of their differentiation competence (Hawkins et al., 2017).

Until recently, stepwise differentiation protocols, mimicking *in vivo* lung mouse lung development have not attempted to capture the step from ventralised anterior foregut progenitors (NKX2-1^+^/SOX2^+^) to distal lung tip progenitors (NKX2-1^+^/SOX2^+^/SOX9^+^) (Nikolić et al., 2017). These human tip progenitors are likely to give rise to all pulmonary lineages, functioning similarly to mouse tip progenitors (Nkx2-1^+^/Sox2^−^/Sox9^+^) (Rawlins et al., 2009a). Recently, SOX2^+^/SOX9^+^ tip progenitor status has been achieved by stepwise hPSC differentiation; these cells can be maintained in relatively pure form for long-term passaging and also undergo spontaneous differentiation (Miller et al., 2017). It will be interesting to understand whether specifically passing through this additional SOX2^+^/SOX9^+^ progenitor phase will improve hPSC differentiation quality, or if the ability to expand this progenitor cell type will increase the quantity of mature lung cells that can readily be obtained.

### Increasing the efficiency of lung differentiation

After the initial studies on lung differentiation from PSCs were reported, subsequent research focused on increasing the efficiency of pulmonary lineage progenitor induction. Highly efficient NKX2-1^+^ (>85%) progenitor induction was achieved by refining the timing of BMP, TGFβ, Wnt and retinoic acid signalling (Huang et al., 2014). One debated question was whether NKX2-1 expression identifies human lung-competent foregut progenitors fitting the current paradigm of mouse lung development. This can be addressed by testing whether hPSC-derived NKX2-1^+^ and NKX2-1^−^ foregut progenitors have equal potential to produce lung lineages. Efforts were thus made to identify cell surface markers that could distinguish NKX2-1^+^ and NKX2-1^−^ foregut cells. Carboxypeptidase M (CPM) was reported as a marker for NKX2-1^+^ pulmonary lineage progenitors by comparing gene expression profiles of *in vitro*-generated anterior foregut cells before and after ventralisation (Gotoh et al., 2014). Sorted CPM^+^ cells were highly NKX2-1 enriched (∼90%) and have been shown to give rise to bronchiolar and alveolar lineages *in vitro* using various different differentiation conditions (Gotoh et al., 2014; Konishi et al., 2016; Yamamoto et al., 2017). Systematic exploration of surface markers for the NKX2-1^+^ population was also achieved using a GFP reporter knocked into the endogenous *NKX2-1* locus (Hawkins et al., 2017). Single-cell RNA-seq of NKX2-1^+^ and NKX2-1^+^/NKX2-1^−^ mixed populations identified *CD47* expression as most highly correlated with *NKX2-1* levels; interestingly *CPM* expression was also highly correlated with *NKX2-1* levels. Combining flow cytometry for CD47^+^ cells with a negative selection marker, CD26 (DPP4), also achieved a high percentage (∼90%) of NKX2-1^+^ enrichment. NKX2-1^−^ cells were discovered to be more prone to non-lung lineage differentiation, including oesophageal, liver and intestinal lineage-like cells. Thus, the *in vitro* differentiation experiments confirmed that NKX2-1^+^ foregut cells are human lung progenitors, as has been indicated by *in vivo* mouse experiments (Minoo et al., 1999; Serls et al., 2005).

Achieving a high induction efficiency, and isolating a pure population, of pulmonary lineage progenitors has been beneficial for downstream differentiation to mature lung cell lineages (Gotoh et al., 2014; Jacob et al., 2017; McCauley et al., 2017). However, there is still work to be done. A recent study used single-cell RNA-seq to analyse the exact lineage specification of differentiated lung cells obtained from a sorted population of NKX2-1^+^ lung progenitors and found that 20-30% of the mature cells obtained were of hepatic or gastric epithelial lineages (McCauley et al., 2018). Biologically, this may reflect the endogenous potential of NKX2-1^+^ foregut progenitors to contribute to multiple organs, contamination of the sorted cell population with NKX2-1^−^ cells, or the *in vitro* derivation of non-lung progenitors that express a low level of NKX2-1. These results highlight that the downstream use of hPSC-derived lung cells may require identification of additional surface markers for selecting pure populations of the mature cells. We should note that surface marker-based purification of NKX2-1^+^ pulmonary progenitor cells is still at an early stage and these methods often rely on reporter lines to faithfully reflect gene expression levels, which may vary in activity between cell lines, or lead to artefacts if the reporters do not behave as expected.

Efforts have also been made to generate specific functional lung lineages more efficiently. For example, ciliated cells have been derived in air liquid interface (ALI)-cultures with Notch signalling inhibition (Firth et al., 2014), a strategy based on mouse developmental biology (Rock et al., 2011b). However, more mature ciliated cells are only generated using 3D culture (Konishi et al., 2016; McCauley et al., 2017). The generation of AT2 cells has also been of great interest because of their stem cell properties. In this vein, alveolar spheroids have been generated by two groups using surface-marker-based enrichment of NKX2-1^+^ lung lineage progenitors with similar growth factor conditions (Jacob et al., 2017; Yamamoto et al., 2017). These SFTPC^+^ spheroids can be expanded long-term and share features of immature alveolar progenitor and mature AT2 cells. Moreover, lamellar bodies can be observed in these SFTPC^+^ cells, which also show the ability to synthesise surfactant-specific lipids, suggesting a mature surfactant processing function.

The differentiation of hPSCs into the various different cell types of the lung provides a platform for drug discovery and possible cell therapy (Box 1). However, it also can help to improve our understanding of human lung development. Wnt, BMP4 and retinoic acid were shown to be the minimal requirements for inducing pulmonary lineage progenitors from hPSCs *in vitro*, a finding that was subsequently confirmed in mice using *ex vivo* culture systems (Serra et al., 2017). Wnt signalling is also important for proximal-distal patterning and alveolar proliferation during mouse lung development (Frank et al., 2016; Li et al., 2005, 2002; Shu et al., 2005). Using *in vitro* differentiation, Wnt signalling has been shown to have a similar role in human lung development, acting by inhibiting proximal, and promoting distal, fate at the NKX2-1^+^ lung progenitor stage (McCauley et al., 2017). During late alveolar differentiation, by contrast, Wnt agonist withdrawal appears to stimulate efficient generation of more mature SFTPC^+^ AT2 cells (Jacob et al., 2017), suggesting that Wnt signalling needs to be tightly regulated at different developmental stages to balance proliferation and differentiation.

### Epithelial-mesenchymal interactions: towards improved *in vitro* differentiation?

There has been increasing interest in understanding the epithelial-mesenchymal interactions that occur during human lung development. The co-culture of ventralised anterior foregut progenitors with foetal lung mesenchyme enhances their differentiation towards bronchiolar and alveolar lineages, illustrating the importance of these cellular interactions. (Gotoh et al., 2014; Yamamoto et al., 2017). However, the actual molecular mechanisms are largely unknown. Interestingly, in some hPSC differentiation methods, mesenchymal cells can be co-derived with epithelial progenitors (Chen et al., 2017; Dye et al., 2016; Dye et al., 2015). It has also been shown that hPSC-derived NKX2-1^+^ epithelial, and associated mesenchymal, cells self-organise into lobular structures, possibly mimicking the tubular structures observed in the developing human foetal lung (Chen et al., 2017). In these experiments, the mesenchymal cells are gradually lost in the cultures; moreover, it has not yet been shown whether the mesenchymal cells obtained are specifically lung mesenchyme and if they directly interact with the neighbouring epithelium. Thus, further optimisation of co-derivation systems as models to dissect epithelial-mesenchymal crosstalk is required.

Another way to achieve relatively robust and consistent lung differentiation is by *in vivo* grafting into the mouse kidney capsule or lung epithelium (Chen et al., 2017; Huang et al., 2014; Miller et al., 2017). hPSC-derived lung organoids have also been cultured on scaffolds implanted into immune-compromised mice, resulting in improved airway epithelial, and mesenchymal, maturation compared with *in vitro* differentiation (Dye et al., 2015; Dye et al., 2016). In general, *in vivo* grafting experiments generate better differentiation compared with *in vitro* models, including co-culture with fibroblasts. This could be due to difficulties in mimicking the multiple factors that contribute to proper lung development *in vivo*, including mechanical forces, extracellular matrix and signals, such as those provided by endothelial cells that co-develop with epithelium or by immune cells that are recruited to the lung during development (Li et al., 2018, 2017; Yamamoto et al., 2007).

Despite these various advances, we should note that cells generated through hPSC-directed differentiation are still rather more like foetal cells in most instances. Hence, understanding late-stage lung development in mouse and other organisms will likely benefit *in vitro* human lung differentiation. Conversely, systematic optimisation of *in vitro* differentiation of hPSCs will also reveal differences with mouse lung development.

## Conclusions and future directions

Human lung developmental studies performed on bona fide human lung tissue are still relatively rare, and much cellular and molecular characterisation remains to be done. However, the efforts of tissue banks, such as the UK Human Developmental Biology Resource (Gerrelli et al., 2015), and focused consortia that make their data available to the research community, such as LungMAP and the proposed Human Developmental Cell Atlas (Behjati et al., 2018), are bringing human studies to the forefront of lung developmental biology. Biologically significant cellular and molecular differences have been identified between mouse and human lungs (Kho et al., 2010; Nikolić et al., 2017) and have highlighted that studying human-specific mechanisms will be essential for understanding disease mechanisms. In the short-term, improved characterisation throughout all stages of human development is needed to better understand the signalling networks and epithelial-mesenchymal crosstalk involved in lung differentiation and morphogenesis. Specific *in vitro* models of the epithelial-mesenchymal cross-talk are also required. For instance, the late-stage vasculature develops alongside the epithelium, and this proximity suggests a crosstalk that has been largely unexplored. In addition, a recent report suggests that mesenchymal changes in β-catenin phosphorylation are conserved between bronchopulmonary dysplasia, which affects neonates, and Idiopathic Pulmonary Fibrosis, which is a chronic adult degenerative disease (Sucre et al., 2018a), further illustrating the importance of understanding the mesenchymal contribution to lung development.

An improved understanding of normal human lung development will facilitate improvements in human *in vitro* experimental systems. Novel methods of growing human embryonic lung cells have recently been developed (Miller et al., 2017; Nikolić et al., 2017; Sucre et al., 2016; Wilkinson et al., 2017). These will allow *in vitro* expansion, and genetic-modification, of scarce human material for functional experiments. However, these methods will always be relatively low-throughput and require validation against actual human lung samples whilst they continue to be optimised. An alternative *in vitro* approach is to differentiate hPSCs to lung fates; such studies have already provided insights into human lung developmental biology. Furthermore, hPSCs have the advantages of limitless supply, ease of genetic manipulation and the possibility of producing large numbers of cells for high-throughput experiments. Similar to the human embryonic lung cells grown *in vitro*, hPSC lung differentiation is still being optimised for the production of pure populations of mature cells and requires continued validation against human lung samples. These differing *in vitro* approaches are largely complementary and can all be adapted for disease modelling and testing regenerative strategies. Further improvements in these models are likely to come from collaborations with bioengineers to include more physiological features such as stretch, gas concentrations and extracellular matrix (Gjorevski et al., 2016; Schneeberger et al., 2017).

As *in vitro* experimental models progress, one challenge to be faced by the field will be the validation of the *in vitro* cultures against human lungs. Adult human lung cells are relatively easy to obtain, as are embryonic samples up to 20 pcw. However, many *in vitro* experiments aim to model late embryonic, or neonatal, lungs. How are these to be validated? The work of the LungMAP consortium in characterising human lungs from 23 pcw to 10 years will help, but is unlikely to be able to collect all of the validation data required. One approach could be to perform complementary experiments in larger mammals whose lungs are more similar to those of humans than of mice. For example, primates have been used to study alveologenesis, bronchopulmonary dysplasia and postnatal lung growth (Fanucchi et al., 2006; Maniscalco et al., 2002). Premature lambs are used as models for BPD (Albertine, 2015), and transgenic pigs are used for modelling cystic fibrosis and now also for fundamental developmental insights (Chen et al., 2018; Rogers et al., 2008). Comparative approaches could be used to determine the core features of neonatal lung cell types of large animals, which could then be used to improve human *in vitro* models. Comparative approaches would also be valuable for identifying core, conserved molecular pathways that may be of potential therapeutic use and even for testing those potential therapies. Moreover, increasing the number of species used for fundamental developmental studies may allow long-standing questions about size control, branching morphogenesis and developmental timing to be addressed.

The current rapid advances in the study of human lung developmental biology make this an excellent time for developmental biologists, geneticists and theorists to work together to identify molecular mechanisms underlying human disease. Numerous genetic variants have now been associated with childhood-onset, and adult-degenerative, lung conditions, and large-scale transcriptional and epigenetic datasets from diseased human lungs are being mined by theorists to identify prospective therapeutic targets. The addition of human lung developmental biology to this mix should allow molecular mechanisms to be validated and rational therapies moved towards the clinic.

## Supplementary Material

Supplementary information
