# Supplementary material for: Human lung development: recent progress and new challenges
Source: Development. 2018 Aug 15;145(16):dev163485. doi: 10.1242/dev.163485 (PMC6124546; doi:10.1242/dev.163485)
Supplement: Supplementary information [file develop-145-163485-s1.pdf]

# Table S1. Summary of published lung differentiation protocols from human pluripotent stem cells

| | **Pulmonary progenitor specification** | | | **Bronchiolar and alveolar lineage specification** | | | | |
|---|---|---|---|---|---|---|---|---|
| **Reference** | **Definitive endoderm (SOX17+/FOXA2+)** | **Anterior foregut endoderm (SOX2+/FOXA2+)** | **Ventralized anterior foregut endoderm (NKX2-1+/SOX2+/TUJ1-/PAX8-)** | **Bronchiolar lineage differentiation** | **Alveolar lineage differentiation** | **Cell types** | **2D or 3D conditions for broncho or alveolar lineage specification?** | **Disease model** |
| Green et al, 2011 | >95% efficiency; Activin A, BMP4, FGF2 for 4 days | ~90% efficiency; Noggin, SB431542 (TGFβ inhibitor) for 2 days | ~37% efficiency; WNT3A, FGF10, FGF7, BMP4, EGF for 4 days | N/A | Kidney graft | *SFTPC* mRNA detected in vitro; SFTPC+ cells in kidney capsule graft | 2D | |
| Mou et al, 2012 | 85%-90% efficiency; RPMI-1640 medium + Activin A, LY284002 (PI3 kinase inhibitor) for 4 days | Up to 50-60% efficiency; A-83-01 (TGFβ inhibitor) and Noggin for 4 days | 10%-30% efficiency; BMP4, FGF2, GSK3iXV (GSK3β inhibitor) for 4 days | Subcutaneous graft in immune-compromised mouse | N/A | Basal cells (but not secretory or ciliated) in mouse graft | N/A | Potential cystic fibrosis |
| Wong et al, 2012 | >85% efficiency; Activin A, WNT3A for 4 days | | >60% efficiency; FGF2, SHH for 5 days | FGF7, FGF10, BMP4 for 6 days; FGF7, FGF10, FGF18 for 4 days; Bronchial epithelial growth media with FGF18 | N/A | Basal cells, ciliated cells, mucin-secreting goblet cells, low club cells | 2D-ALI (Air Liquid Interface) | Cystic fibrosis |

Development • Supplementary information

| | | | | | | | |
|---|---|---|---|---|---|---|---|
| Huang et al, 2104 | Wnt3a, BMP4, Y-27632 (ROCK inhibitor) for 1 day; Activin A, BMP4, FGF2 for 3 days | Dorsomorphin (BMP inhibitor), SB431542 (TGFβ inhibitor) for 1 day; IWP2 (Wnt inhibitor), SB431542 (TGFβ inhibitor) for 1 day | Up to > 85% efficiency; CHIR99021 (GSK3β inhibitor), FGF10, FGF7, BMP4, RA for 9 days | CHIR99021 (GSK3β inhibitor), FGF10, FGF7 for 10 days; CHIR99021 (GSK3β inhibitor), FGF10, FGF7,Dexamethosone, cAMP, IBMX (DCI) for >3 weeks, or kidney capsule graft | In vitro culture: ciliated cells, mucin-secreting goblet cells, club cells, basal cells, SPB+ AT2 cells, AT1 cells<br>Kidney graft: basal, ciliated, club, goblet cells observed. AT1 and AT2 cells. Smooth muscle cells and cartilage, mesodermal cells. | 2D | |
| Gotoh et al, 2014 | >80% efficiency; Sodium butyrate, Activin A, CHIR99021 (GSK3β inhibitor) for 6 days | >88% efficiency, Noggin, SB431542 (TGFβ inhibitor) for 4 days | ~60-70% efficiency; BMP4, RA, CHIR99021 (GSK3β inhibitor) for 4 days | Sorted CPM+ cells in DCI, FGF7 with 17pcw fetal lung fibroblasts for 10 days | Ciliated cells, goblet cells, AT1 and AT2 cells | 3D co-culture | |
| Firth et al, 2014 | ~60% efficiency, WNT3A, Activin A for 1 day; Activin A for 1 day | | ~50% efficiency, Noggin, SB431542 (TGFβ inhibitor) for 1 day; BMP4, SB431542 (TGFβ inhibitor) for 4 days | Hydrocortisone, T3, Epinephrine, RA, Phosphorylethanolamine, Ethanolamine for >3 weeks | Ciliated cells, club cells, goblet cells, CD90+ mesenchymal cells | 2D-ALI | |

Development • Supplementary information

| | | | | | | | | |
|---|---|---|---|---|---|---|---|---|
| Dye et al., 2015 | Activin A for 4 days | Noggin, SB431542 (TGFβ inhibitor), FGF4, CHIR99021 (GSK3β inhibitor), SAG (Smoothened agonist) for 6 days. (Foregut spheroids are induced and can be pinched off and embedded in Matrigel) | Pinched off spheroids in Matrigel with FGF10 for up to 65 days with repeated passaging | N/A | N/A | Proximal and distal lung epithelial structures with associated mesenchyme cells | 3D | |
| Konishi et al, 2016 | Sodium butyrate, Activin A,CHIR99021 (GSK3β inhibitor) for 6 days | Noggin, SB431542 (TGFβ inhibitor) for 4 days | BMP4, RA, CHIR99021 (GSK3β inhibitor) for 4 days | Sorted CPM+ cells in FGF10, CHIR99021, FGF7, DAPT for 2 weeks; CHIR99021, FGF10 in 3D for 2wks; PneumaCult with DAPT for 4 weeks | N/A | Ciliated cells, club cells, basal cells, goblet cells, neuroendocrine cells | 3D-ALI | |
| Dye et al., 2016 | Activin A for 4 days | Noggin, SB431542 (TGFβ inhibitor), FGF4, CHIR99021 (GSK3β inhibitor), SAG (Smoothened agonist) for 6 days. Foregut spheroids are induced and can be pinched off and embedded in Matrigel | Pinched off spheroids in matrigel with FGF10 for 15 days | Spheroids grown on scaffolds and implanted into immune-compromised mice | N/A | Goblet cells, basal cells, ciliated cells, club cells, neuroendocrine cells, associated mesenchyme including cartilage and fibroblasts | 3D + scaffolds + in vivo | |

Development • Supplementary information

| | | | | | | | | |
|---|---|---|---|---|---|---|---|---|
| Hawkins et al, 2017 | STEMdiff Definitive Endoderm Kit for 3 days | Dorsomorphin (BMP inhibitor), SB431542 (TGFβ inhibitor) for 3 days | ~20-30% efficiency, CHIR99021 (GSK3β inhibitor), BMP4, FGF10, FGF7, RA for 9 days | Sorted NKX2-1-EGFP+, or CD47hi,CD26low, in CHIR99021 (GSK3β inhibitor), FGF10, FGF7 for 7 days; CHIR99021 (GSK3β inhibitor), FGF10, FGF7, DCI for >3 weeks | | Airway and alveolar lineages | 3D | |
| McCauley et al, 2017 | STEMdiff Definitive Endoderm Kit for 3 days | Dorsomorphin (BMP inhibitor), SB431542 (TGFβ inhibitor) for 3 days | ~35% efficiency, CHIR99021 (GSK3β inhibitor), BMP4, FGF10, FGF7, RA for 9 days | Sorted NKX2-1-GFP+ in FGF2, FGF10, DCI for >2 weeks | N/A | Airway: ciliated cells, club cells, basal cells, goblet cells | 3D | cystic fibrosis, swelling organoids |
| Jacob et al, 2017 | STEMdiff Definitive Endoderm Kit for 3 days | Dorsomorphin (BMP inhibitor), SB431542 (TGFβ inhibitor) for 3 days | CHIR99021 (GSK3β inhibitor), BMP4, RA for 9 days | N/A | Sorted NKX2-1-EGFP+ or CD47hiCD26low in CHIR99021 (GSK3b inhibitor), FGF7, DCI for 2 weeks; FGF7 for 1 week; CHIR99021 (GSK3β inhibitor)+DCI for 1 week | AT2 cells | 3D | Genetic alveolar disease (*SFTPB* mutation) |

Development • Supplementary information

| | | | | | | | | |
|---|---|---|---|---|---|---|---|---|
| Yamamoto et al, 2017 | Sodium butyrate, Activin A, CHIR99021 (GSK3β inhibitor) for 6 days | Noggin, SB431542 (TGFβ inhibitor) for 4 days | ~85% efficiency, BMP4, RA, CHIR99021 (GSK3β inhibitor) for 4 days | N/A | Sorted CPM+ in CHIR99021 (GSK3β inhibitor), FGF10, FGF7, DAPT for 1 week; FGF7, DCI co-culture with human fetal lung fibroblasts or without in CHIR99021 (GSK3β inhibitor), SB431542 (TGFβ inhibitor), FGF7, DCI | AT2 and AT1-like cells | 3D±co-culture | Drug toxicology studies (recapitulating enlarged lamellar body phenotypes) |
| Chen et al, 2017 | BMP4, Y-27632 (ROCK inhibitor) for 1 day; BMP4, Y-27632 (ROCK inhibitor), Activin A, FGF2 for 3 days | Noggin, SB431542 (TGFβ inhibitor) for 1 day; IWP2, SB431542 (TGFβ inhibitor) for 1 day | ~50% efficiency, CHIR99021 (GSK3β inhibitor), BMP4, FGF10, FGF7, RA ~2wks (spheres float off and can be expanded in suspension, or transferred to Matrigel). | CHIR99021 (GSK3β inhibitor), FGF10, FGF7 BMP4, RA, or kidney graft | | In vitro: SPB+, SPC+, ABCA3+ AT2 cells, goblet cells, CD90+VIM+ mesenchymal cells Kidney graft: goblet cells, basal cells, ciliated cells, club cells, neuroendocrine cells, AT2 cells, AT1 cells, SMA+ mesodermal cells | 3D | |

Development • Supplementary information

| | | | | | | | |
|---|---|---|---|---|---|---|---|
| Miller et al, 2018 | ActivinA for 4 days | Noggin, SB431542 (TGFB inhibitor), FGF4, CHIR99021 (GSK3B inhibitor), SAG (Smoothened agonist) for 6 days. (Foregut spheroids are induced and can be pinched off and embedded in Matrigel) | Pinched off spheroids in Matrigel | FGF7, CHIR99021 (GSK3B inhibitor), RA for 2-10 weeks; serial needle passage; FGF7 or engrafting in injured mouse lung epithelium | Club cells, neuroendocrine cells, AT2 cells, AT1 cells in vitro; club cells, goblet cells, ciliated cells in mouse injured lung epithelium engraftment | 3D | |

Development • Supplementary information
